# A Supplement with Ribes Nigrum, Boswellia Serrata, Bromelain and Vitamin D to Stop Local Inflammation in Chronic Sinusitis: A Case-Control Study

**DOI:** 10.3390/jcm12082929

**Published:** 2023-04-18

**Authors:** Pietro De Luca, Luca D’Ascanio, Cristina Cingolani, Gino Latini, Egle Grigaliute, Paola Di Mauro, Massimo Ralli, Ignazio La Mantia, Arianna Di Stadio

**Affiliations:** 1 Department of Otolaryngology, San Giovanni-Addolorata Hospital, 00100 Rome, Italy; 2 Otolaryngology Department, AORMN (Azienda Ospedali Riuniti Marche Nord), 61032 Fano, Italy; 3 Department GF Ingrassia, Otolaryngology, University of Catania, 95131 Catania, Italy; 4 Organ of Sense Department, University La Sapienza, 00185 Rome, Italy

**Keywords:** chronic sinusitis, treatment, Boswellia, Vitamin D, nasal hyperemia, rhinorrhea

## Abstract

Although chronic sinusitis widely affects the adult population, the treatments currently used did not always satisfactorily solve the symptoms. Traditional therapy with steroids and antibiotics presents risks and benefits and the new drugs, i.e., monoclonal antibody, are valid solutions despite being quite expensive. Natural molecules could be a valid treatment that combines good efficacy and low price. We conducted a case -control study to evaluate the benefit of an oral supplement with Ribes nigrum, Boswellia serrata, Bromelain and Vitamin D on chronic sinusitis symptoms. 60 patients were randomly assigned to one of the three groups: control using nasal steroids only, treatment 1 using nasal steroid and 1 dose of the oral supplement for 30 days and treatment 2 in which patients used nasal steroid and two oral supplement doses daily for 15 days. Conditions of the nasal mucosa and a blood sample (WBC, IgE and CRP) were analyzed at T0, T1 (15 days after treatment) and T2 (30 days after treatment. Patients treated with the supplement improved their nasal findings (hyperemia of mucosa and rhinorrhea) with statistically significant differences from the control. Our preliminary data suggest that the addition of supplement containing Ribes nigrum, Boswellia serrata, Vitamin D and Bromelain to the traditional local therapy (nasal spray with cortisone) can be a supporting therapy to modulate the local inflammation in the nose in patients affected by chronic sinusitis.

## 1. Introduction

The term sinusitis refers to the inflammation of the sinus or nasal ostium; if the condition lasts by four weeks is considered acute, while, if it persists over 12 weeks is defined as chronic. 

Chronic sinusitis (CS) can have different forms as (1) without nasal polyps; (2) with nasal polyps; (3) allergic fungal rhinosinusitis and others [1,2].

Several factors concur with the onset of CS [3]. The nasal microbiota, which represents the common flora present in the nose and nasal sinus, can be modified by a viral infection or other bacteria not commonly resident in the nose, determining, once acute sinusitis is resolved, a chronic and persistent local inflammation [4]. Other conditions including airborne irritants, cigarette smoke or other toxins and congenital or acquired immune disorders [4] can also negatively impact by changing the nasal environment and reducing the local immune answer [5,6]. Moreover, CS can be caused by a host’s allergy (excessive answer to common element as dust); this condition induces a constant inflammation of the mucosa followed by mucosal hyperplasia [5,6]. Finally, chronic sinusitis is multifactorial in nature and can include infectious, inflammatory, or structural factors [5,6].

The treatment of sinusitis is chosen being focused on the trigger event [7]. In case of allergy, nasal steroids with/without antihistamines are used [8]; if the sinusitis is due to an infection, antibiotic therapy associated with nasal spray or aerosol is prescribed [9]. Finally, in case of fungal infection, a combination between nasal steroids and antibiotics is considered first, followed by anti-mycotic treatment in case of unsuccess [10].

Treatments aim at reducing local inflammation (steroids), improving the nasal environment by stopping bacterial aggression (antibiotics), and modulating the immune response to fight the excessive response (antihistamine).

However, these treatments can present local and systemic adverse events; for example, local steroid increase the risk of epistaxis and smell alteration [11], antibiotics can cause antibiotic resistance [12] and antihistamines may induce excessive somnolence [13].

Thanks to the increased availability of high-quality nutraceuticals, these molecules, i.e., vitamin D, can be used to stimulate the immune response both systemically [14] and locally [15], and to modulate the immune system [16]. In addition, there are some natural elements [17] with anti-inflammatory abilities, i.e., bromelain [18], that can be helpful in fighting nasal inflammation [17].

Therefore, a supplement containing both immune-modulatory and anti-inflammatory natural molecules could be a useful tool to ameliorate nasal conditions, especially in patients with chronic conditions.

The aim of this prospective case-control study was to evaluate the effects of a nutraceutical (Flogostop Forte^®^ Humana^®^ Italia S.p.A.) containing Ribes nigrum, Bromelain, Boswellia serrata (Casperome^®^) and Vitamin D on the improvement of symptoms in patients with sinusitis, and to speculate how it modulates the inflammatory and immune pathways in these patients.

## 2. Materials and Methods

This study was conducted at the Departments of Otolaryngology of “Santa Croce” Hospital (Fano, Italy) from January to November 2022. All procedures were approved by the local Institutional Review Board (IRB) and conducted in accordance with the ethical principles outlined in the Declaration of Helsinki. The participants signed a written, informed consent to authorize enrollment in the study.

Sixty patients were recruited (18 women and 42 men; age 52.5 + 9.9). Twenty patients were in TG1 (16 men and 4 women; age 51.5 + 7.7), 20 in TG2 (14 men and 6 women; age 52.1 + 11.4) and 20 in the control group (12 men and 8 women; 53.8 + 11).

All the groups were homogeneous for baseline conditions (*p* < 0.05). None of the patients had comorbidities and none of them was a smoker.

### 2.1. General Information and Randomization

The nutraceutical used in the study was an oral solution available on the market commercial name Flogostop Forte^®^ produced by Humana^®^ Italia S.p.A; the supplement contains in 4 gr sachet the following elements Vitamin D (15 microgram, 600 U.I.), Boswellia Casperome^®^ (125 mg), Bromelain (200 mg), and Ribes nigrum (300 mg).

All patients were screened by a doctor who filled in the patients’ clinical records and did the randomization using a computer; then, a different physician (without any information on the patient assigned) performed all the clinical investigations at each follow-up. The use of two different doctors guaranteed a single-blinded analysis.

After randomization, the patients were assigned to one of three groups: Control Group (CG), Treatment Group 1 (TG1-Flogostop 1), or Treatment Group 2 (TG2-Flogostop 2). The patients in both treatments group were treated by Flogostop Forte^®^ (TG1; 1 sachet Flogostop Forte^®^/die for 4 consecutive weeks. TG2; 2 sachet Flogostop Forte^®^/die for 2 consecutive weeks) in combination with the standard treatment, instead, CG was only treated with standard treatment for 4 consecutive weeks.

The traditional treatment consisted of corticosteroids administered through nasal spray [mometasone 2 puffs twice a day]. Patients already included in the study, who had a fever and need antibiotic treatment during the follow-up period, were excluded. All patients started the treatment within two days after the first clinical consultation.

Three time points were identified: T0 = before treatment (baseline), T1 = two weeks after treatment with Flogostop Forte^®^, and T2 = four weeks after treatment with Flogostop Forte^®^.

### 2.2. Inclusion and Exclusion Criteria

Patients suffering from chronic sinusitis without nasal polyps in accordance with clinical EPOS classification (children > 12yo, adolescents and adults), not treated in the last 6 months with antihistamine therapy and not under-treatment with corticosteroids (topic or systemic) were enrolled in the present study.

Exclusion criteria were: patients affected by maxillary sinusitis already under treatment at the moment of the enrollment; patients who underwent endoscopic surgery in the last 12 months; patients under treatment with systemic antihistamine or corticosteroids; patients affected by asthma; patients treated by radio (RT) +/− chemotherapy (CH) on head and neck district in the last 36 months; patients under treatment for cancer; patients with a serious neurological condition; patients with known immunodeficiency (i.e., AIDS); patients affected by purulent sinusitis with fever; patients under-treatment with anticoagulant (i.e., warfarin) and aspirin.

### 2.3. Clinical Evaluation

The patients were evaluated by an otolaryngologist with >10-year experience. The physician evaluated the condition of the patient’s nasal mucosa using a flexible fiberoptic endoscope (Karl Storz, Tuttilingen, Germany) and an Olympus CV-170 camera (Olympus, Shinjuko, Tokyo, Japan). The clinical aspect of the nasal mucosa was photo-recorded and then, based on the finding a score of 0 in case of normal color and 1 in case of hyperemia was assigned; to reduce inter-operator variability in the evaluation of the condition of nasal mucosa all patients were evaluated by two senior physicians (CG and GL) with over twenty years of experience to assign the correct score. If a patient presented spontaneous rhinorrhea, we assigned score 1, in absence score 2; this sign was evaluated only as “present or not” without quantifying it. Blood tests, including white cell counts, serum IgE, and C-Reactive Proteine (CPR) were performed at the three follow-ups (T0, T1, and T2); the sinusitis questionnaire, translated into Italian, was used to evaluate the general condition of the patients at the three follow-ups (T0, T1, and T2) [19].

The following data were collected for each patient: gender, age, voluptuary habit as use of recreational drugs, major disease, therapeutic treatment.

### 2.4. Statistical Analysis

One of the authors (ADS) certified in biostatistics participated in the study and in the statistical design. Both within and between analysis were performed. Chi-square was used to analyze the differences in the nasal endoscopic findings in CG, then in TG1 and finally in TG2 at the three different follow-ups. The same test was performed to evaluate the difference in the nasal finding between the groups (TG1, TG2, and CG) at the three time points (T0, T1, and T2). One-way ANOVA was done to evaluate the variance of blood tests with white cells counts, IgE, CPR, and sinusitis questionnaires. Holm–Bonferroni (HB) ad hoc test was performed for each one-way ANOVA. *p* value was considered statistically significant < 0.05. The statistical analysis was performed by STATA^®^.

## 3. Results

### 3.1. Within Group Analysis

#### 3.1.1. Treatment Group 1

**Figure 1:**
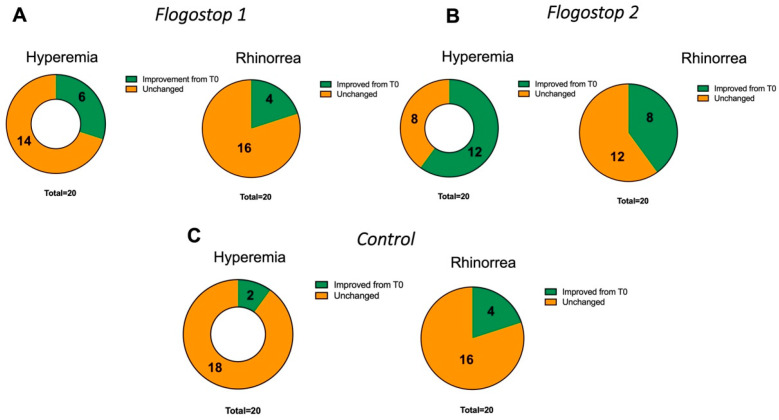
Clinical Findings. (**A**) Improvement in nasal hyperemia and rhinorrhea in TG1 comparing the baseline with T2, 30 days after. In this group, the supplement has been suspended at T1 (15 days after use). (**B**) Comparison of nasal findings in TG2 after 30 days of treatment (T2). (**C**) Results obtained in term of nasal improvement using exclusively nasal cortisone spray.

Statistically significant variations were observed (Chi-Square: *p* = 0.02) analyzing the improvement of nasal hyperemia after treatment at T2. (Figure 1A).

No statistically significant improvement was observed in the rhinorrhea parameter (Chi-square: *p* = 0.2) (Figure 1A).

**Figure 2:**
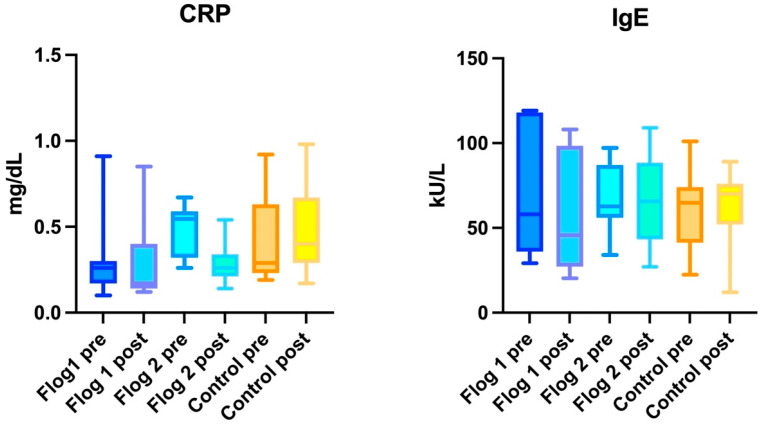
The images show the measurements of C-Reactive Protein (CRP) and IgE in TG1, TG2 and control group comparing the baseline with the end of the treatment. The vertical line indicates standard deviation.

No statistically significant variances were observed at the three different follow-ups either in CRP (ANOVA: *p* > 0.05) or IgE levels (ANOVA: *p* > 0.05) (Figure 2).

**Figure 3:**
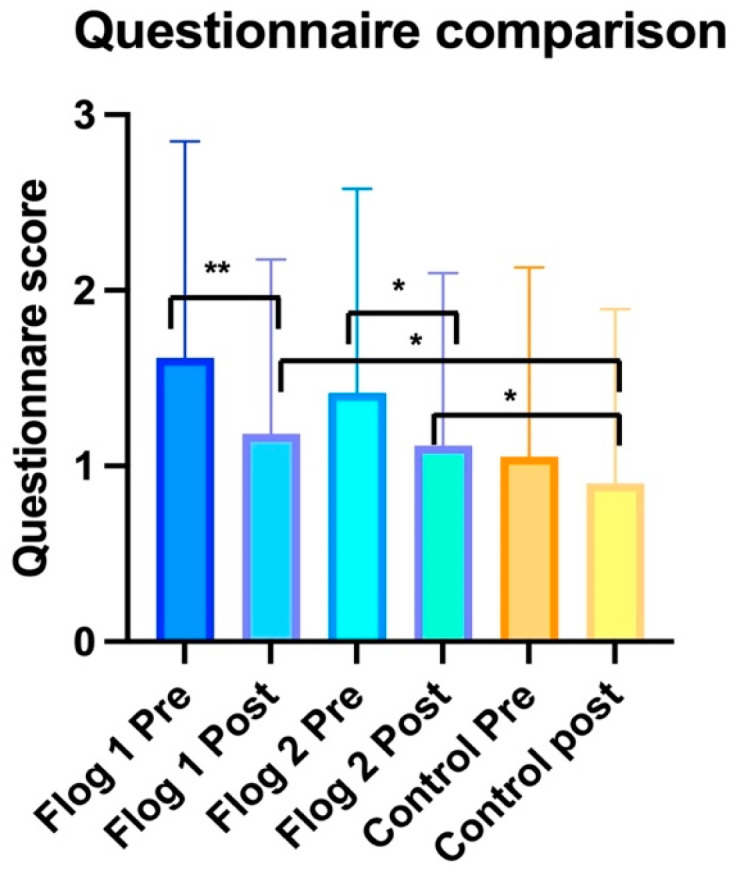
The graph shows the results of the questionnaire. “**” indicates *p* < 0.01 and “*” *p* < 0.05. The vertical line shows standard deviation. We identified statistically significant differences in the treatment groups (Flog 1 and Flog 2) before and after treatment but none in the control group. The graph also shows the differences of questionnaire scores between treatment groups and control post-therapy.

A statistically significant improvement in the score of the questionnaire was observed before and after treatment (ANOVA: *p* < 0.0001). In particular, the improvement was observed between T0 and T1 (BH: *p* < 0.01) and T0 and T2 (BH: *p* < 0.01). No statistically significant differences were observed between T1 and T2 (Figure 3).

#### 3.1.2. Treatment Group 2

Statistically significant variations were observed (Chi-Square: *p* = 0.007) analyzing the improvement of nasal hyperemia after treatment at T2 (Figure 1B). A statistically significant improvement was observed in the rhinorrhea parameter (Chi-square: *p* = 0.04) (Figure 1B).

No statistically significant variances were observed at the three different follow-ups either in CRP (ANOVA: *p* > 0.05) or IgE levels (ANOVA: *p* > 0.05) (Figure 2).

A statistically significant improvement in the score of the questionnaire was observed before and after treatment (ANOVA: *p* < 0.0001). In particular, the improvement was observed between T0 and T1 (BH: *p* < 0.05) and T0 and T2 (BH: *p* < 0.01). Statistically significant differences were observed between T1 and T2 (HB: *p* < 0.05) (Figure 3).

#### 3.1.3. Control Group

No statistically significant variations were observed (Chi-square: *p* = 0.3) analyzing the improvement of nasal hyperemia after treatment at T2 using the nasal spray only (Figure 1C).

No statistically significant improvement was observed in the rhinorrhea parameter Chi Square: *p* = 0.2) (Figure 1C).

No statistically significant variances were observed at the three different follow-ups either in CRP (ANOVA: *p* > 0.05) or IgE levels (ANOVA: *p* > 0.05) (Figure 2).

No statistically significant improvement in the score of the questionnaire was observed before and after treatment (ANOVA: *p* > 0.05) (Figure 3).

### 3.2. Between Groups Analyses

The groups did not show statistically significant differences at the baseline observations (ANOVA: *p* > 0.05).

**Figure 4:**
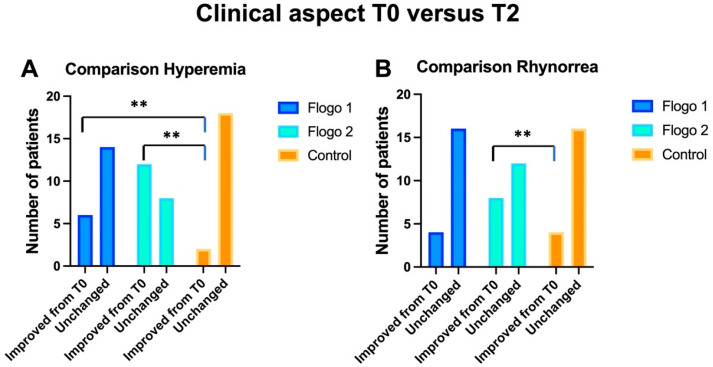
Comparison of clinical aspects between the three groups in term of improvement at the end of the treatment. (**A**) shows the changes of the color of nasal mucosa (hyperemia) and (**B**) the improvement of nasal secretion (reduction of rhinorrhea) after treatments. “**” indicates *p* < 0.01.

Statistically significant differences comparing the hyperemia of nasal mucosa were observed between CG and TG1 and TG2 (Chi-square: *p* = 0.003) at T2 (Figure 4A).

Statistically significant differences were observed comparing rhinorrhea symptoms between CG and TG2 (Chi-square: *p* = 0.001). No statistically significant differences were instead observed between CG and TG1 (*p* = 0.5) (Figure 4B).

No statistically significant variances were observed at the three different follow-ups either in CRP (ANOVA: *p* > 0.05) or IgE levels (ANOVA: *p* > 0.05) analyzing CG and TG1 and TG2.

Statistically significant differences in the score of the questionnaire were observed between CG and treatment groups (ANOVA: *p* < 0.001). The differences were observed between CG and TG1 at T1 (BH: *p* < 0.05) and T2 (BH: *p* < 0.05), and between CG and TG2 at T1 (BH: *p* < 0.05) and T2 (BH: *p* < 0.05) but none between the three groups at the baseline (*p* > 0.05) (Figure 3).

## 4. Discussion

Our study showed that the addition of supplement containing Ribes nigrum, Boswellia serrata (Casperome^®^), Vitamin D and Bromelain to the traditional local therapy (mometasone nasal spray) can be a supporting therapy to modulate the local inflammation in the nose in patients affected by chronic sinusitis as showed by the improvement of the nasal findings; the use of supplement independently from the posology (two sachets or one sachet) used was able to reduce the local hyperemia. The presence of rhinorrhea (excessive nasal secretion of mucus) was reduced only by using the supplement for longer time (1 month).

The “within” analyses, which compared the variances in each singular group (TG1, TG2 and CG) before and after treatment, looking at the investigated nasal findings (mucosal hyperemia and rhinorrhea) showed no statistically significant variance for both conditions in the control groups comparing the data at baseline (T0) and T2. On the opposite, the addition of the supplement to the standard treatment allowed to reduce (with statistically significant value) the nasal hyperemia in TG1 and TG2. For TG1, the short treatment at a higher dose (one sachet two times a day) improved the conditions of nasal mucosa after two weeks of use (T1) and its benefic effect persisted even after tapering it at 30 days follow-up (T2). Even TG2 showed a reduction of nasal hyperemia comparing T0 and T2; in addition, the use of a single dose daily for an extended time (30 days) allowed the reduction of rhinorrhea. The latter, is caused by the excessive activation of the nasal glandular apparatus [20] due to the inflammation of nasal mucosa [20]; the mucosa hyperproduce mucus, which contains anti-bacterial substance, to fight the origin of the nasal inflammatory process [21]. The extension of the treatment for more than 15 days could have substantially reduced the local inflammation by stopping the excessive activation of the nasal glandular apparatus [20,21].

Comparing the nasal findings between the three groups, the use of the treatment, both in the short and long term, was always able to determine a statistically significant improvement of the nasal symptoms and signs when compared with the CG.

Based on these current findings, we speculate that the supplement could have improved local inflammation (hyperemia and rhinorrhea) for different reasons related to its components. Bromelain has immunomodulatory effects and antibacterial properties; these two characteristics in chronic sinusitis allow us to (i) ameliorate the local immune response with reduction of nasal inflammation, (ii) reduce the secretion of pro-inflammatory agents during rhinitis, (iii) reduce mucus secretion and (iv) kill microbes responsible of the recurrent chronic nasal inflammation [22,23].

Vitamin D is a known immune-stimulating and immune-modulating element and the efficacy of supplements containing this vitamin to fight infection and inflammation of the upper respiratory tract has been recently shown in human clinical trials. [24]. A recent meta-analysis performed by Li et al. identified a deficit of this vitamin in a patient with chronic sinusitis [25] suggesting the supplementation with vitamin D as possible useful treatment. Our results confirm that vitamin D can be beneficial to reduce nasal inflammation in patients affected by chronic sinusitis [15].

Boswellia inhibits 5-lipoxygenase (5-LO), including 5-hydroxyeicosatetraenoic acid (5-HETE) and leukotriene B4 (LTB-4) that cause chemotaxis and vascular permeability [26]; in particular, the inhibition of 5-LO may reduce the inflammation in the nasal mucosa and consequently, the secretion of excessive mucus as we observed in this study. The nutraceutical we used contained Boswellia Casperome^®^ based on a patented method able to improve the bioavailability of Boswellia ameliorating the efficacy of the product [27].

Ribes nigrum (Blackcurrants) has potent antioxidant, antimicrobial, and anti-inflammatory properties [28]. This element inhibits the M1 pro-inflammatory phenotype of macrophages [29], reducing the local inflammation and inhibiting the recurrence of nasal symptoms. Furthermore, Ribes nigrum inhibits the replication of a series of viruses, for example, influenza viruses, which sustain the chronicity of naso-sinusal inflammation [30].

**Figure 5:**
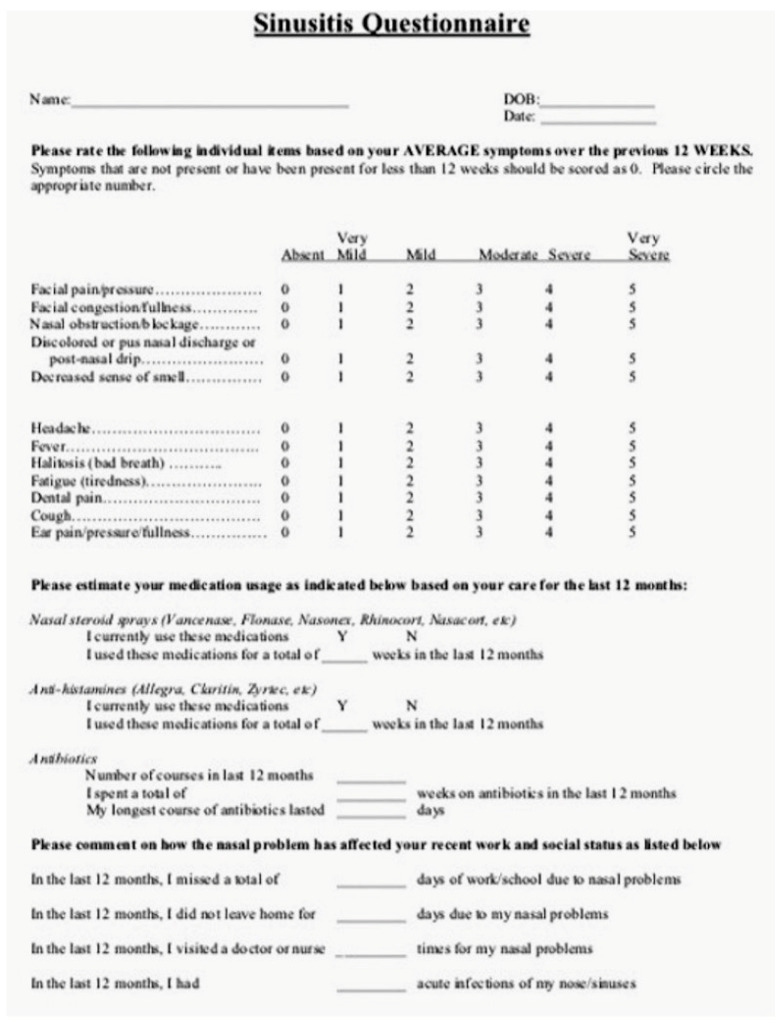
Questionnaire used to screen the patients during the study and to evaluate the clinical variation obtained after the treatments.

In addition to the improvement of the nasal findings, we observed that all patients who used the supplements improved their scores in the self-administered questionnaire. The questionnaire (Figure 5) contains a series of questions that are related to inflammation, pain, and general health. The improvement (*p* < 0.01) of the scores, which was observed both within the group (TG1 and TG2) at the different follow-ups and between groups (comparing CG with the treatment groups), indicated a systemic effect of the supplement, although it was not represented by the statistically significant variance of the CRP and IgE.

The combination of these molecules has already been tested for treating inflammation and infection of the upper respiratory tract in children [30]. The authors showed both the safety and efficacy of the compound. In fact, in the study, the children improved both auditory and respiratory functions and none of them presented neither clinical allergic reaction, for example, urticaria, or changes in the immune answer at cellular levels (normal IgE and white cells) [31].

The authors stated that a combination of anti-inflammatory and anti-viral elements allowed to reduce the inflammation and the recurrent infection in the nose; the reduction of the adenoids volume, because of the previously mentioned effects, freeing the rhynopharynx has improved the auditory functions after 35 days of use [31].

The same mechanism is applicable to chronic sinusitis because both local inflammation and repetitive infections cause the persistence and worsening of the symptoms; the stop of viral replication and the reduction of the local inflammation into the nose [31] allowed to our patients, exactly as the previous study, to improve the clinical symptoms.

*Study limitations*: The main limitation of this study is the small sample size; a larger sample is necessary to confirm our results. As second, we did not stratify the population for age, and some differences in terms of efficacy could be observed comparing young and elderly. Thirdly, this study is based on clinical findings and a self-questionnaire so the local effects of the supplement could be only speculative; additional studies evaluating the inflammatory parameters in the local site (e.g., through nasal brushing) are necessary to fully understand the effect of the supplement. Finally, despite randomization, the baseline scores of the questionnaire in the three groups were different (but not statistically significantly different), and this might impact the results despite considering both within and between variances.

## 5. Conclusions

This study showed that the addition of a supplement with Ribes nigrum, Boswellia serrata (Casperome^®^), Bromelain and Vitamin D to the standard local treatment in chronic sinusitis can improve the clinical findings, first by reducing the inflammation of nasal mucosa, then improving the general symptoms correlated with this disease. The use of the supplement, despite a different efficacy based on the different posology used, was always a benefit when compared with the standard therapy.

## Data Availability

Data are available under request to the corresponding author.
